# Trade-offs and synergies when balancing economic growth and globalization for sustainable development goals achievement

**DOI:** 10.1038/s41598-025-93360-3

**Published:** 2025-03-13

**Authors:** Imre Fertő, Gábor Harangozó

**Affiliations:** 1 Corvinus University of Budapest, Fővám tér 8, Budapest, 1093 Hungary; 2 HUN-REN Centre for Economic and Regional Studies, Tóth K. u. 4, Budapest, 1112 Hungary

**Keywords:** Sustainable development, SDG Index, Globalization, Club convergence, Environmental economics, Sustainability

## Abstract

This study investigates the complex relationships between globalization, economic growth, urbanization, and ecological footprint in the context of advancing the United Nations Sustainable Development Goals (SDGs). Employing a club convergence framework, we evaluate global SDG Index from 2000 to 2023 for 149 countries with 3212 observations, identifying five converging clubs and one non-converging group. Our analysis demonstrates that higher GDP per capita and various dimensions of globalization positively impact SDG outcomes, whereas rapid urbanization and expansive ecological footprints exert negative influences. This research highlights the critical need for tailored policy interventions that address the distinct challenges encountered by different country clusters to bolster sustainable development efforts. Our findings reveal the multifaceted nature of sustainable development, indicating that economic growth and globalization can support SDG advancement if their detrimental effects are effectively mitigated. The study offers valuable insights for crafting national and global strategies to expedite progress towards the SDGs, emphasizing the importance of harmonizing economic, social, and environmental priorities.

## Introduction

The sustainability challenge now more than ever encompasses the realms of society, environment, and economy. In response, the United Nations established 17 Sustainable Development Goals (SDGs) in 2015, aiming to achieve 169 targets by 2030^1^. Despite global endorsement from 193 countries^2,3^, midway through the targeted period in 2023, progress has been deemed insufficient^4^, with only 15% of targets on track, while 48% are moderately or severely off track, and 37% show no progress at all, indicating stagnation or regression.

Globalization, increasingly interconnecting nations, regions, and communities, presents a dual role in advancing the UN SDGs. On one hand, it promotes efficient resource allocation supporting economic and environmental goals^5,6^, facilitates knowledge sharing for environmentally sound technologies^7^ bridges the digital divide through enhanced education and connectivity^8^ and improves access to medical products and services, particularly in developing countries^9^. Conversely, globalization exacerbates sustainability challenges through increasing consumption and material footprint^10^, surpassing planetary boundaries^11^, and by contributing to environmental degradation due to increased transportation needs^12^. Additionally, the anticipated digital gap reduction remains elusive^13,14^ and globalization often diminishes ecological and cultural diversity^15,16^.

Although extensive literature explores globalization’s complex impacts on sustainability, a comprehensive evaluation of its role in overall SDG progress remains limited. Existing research typically focuses on specific sustainability aspects, like resource overuse^17^, waste^18^ and pollution issues^19^, biodiversity^20^ or local communities^21^ rather than a holistic assessment over an extended period.

Economic growth and income also present complex, sometimes contradictory, impacts on sustainability and the UN SDGs. Proponents of green or positive growth argue that economic expansion can align with sustainability goals, positing that social and environmental objectives can be achieved without fundamentally altering the growth paradigm^22,23^ Indeed, even the UN SDG framework itself can be assessed as a positive growth approach, implicitly assuming the possibility to achieve development in all of the economic, environmental and social domains^24^. Critics, however, contend that true sustainability cannot be achieved within the existing growth paradigm^25^, advocating for degrowth^26,27^ or zero growth^28,29^ (or steady-state economics) to resolve underlying social and environmental issues^30,31^. The environmental Kuznets-curve hypothesis^32^, suggesting an inverse U-shaped relationship between GDP per capita and environmental indicators, has been empirically supported in areas, such as carbon-dioxide emissions^33,34^, energy consumption^35^ or health^36^.

Urbanization impacts sustainability significantly. According to World Bank data and forecast^37^, today 56% of world population live in urban areas, tend to increase to 70% by 2050. This trend poses direct sustainability challenges related to urban infrastructure and transportation^38,39^, and indirectly influences economic structures through urbanization^40,41^.

The ecological footprint measuring the biologically productive land and sea area required to support consumption levels^42^ highlights the unsustainability of current consumption trends^43,44^. Balancing ecological footprint with biocapacity involves improving ecosystem service efficiency, reducing global population, or decreasing per capita consumption^45^:

A detailed quantitative analysis of globalization, income, urbanization, and ecological footprint on sustainability, along with country classifications based on these factors, can provide valuable insights for tailored policy interventions. Comparing impacts across different country groups can reveal patterns that inform the development of national and global measures to expedite SDG progress. Although the SDGs target the global community, significant disparities in progress exist among countries^46,47^ raising questions about convergence trends over time.

To contribute to this gap, we analyze whether SDG progress converges using the club convergence framework^48,49^ based on a specific clustering algorithm^50^. In addition, we investigate key factors driving the club convergence in SDG progress. This is a novelty value of this study, there seem to be no such works previously in this framework and the SDG context. The only source^51^ we found related to the SDG framework had a very specific focus on corruption. Other club convergence approach works addressed specific areas of sustainability, such as ecological footprint^52^ or environmental emission constraints^53^.

Based on this gap, the motivation behind this study is to better understand the specific roles of globalization and other determinants of sustainability that can help policymakers to fine-tune strategies to overcome the specific challenges faced by different groups of countries. Furthermore, the findings aim to contribute to the broader discourse on how economic, social, and environmental priorities can be harmonized to promote sustainable development.

The study addresses the following research questions. Are countries converging in their sustainability efforts toward the SDGs and what patterns of convergence or divergence emerge across different groups of countries? What roles do globalization, economic growth, urbanization, and ecological footprint have in shaping SDG performance at a national level?

The UN uses numerous indicators for the assessment of the 17 goals and 169 targets, but there is no one comprehensive UN indicator quantifying overall progress of countries. We use the SDG Index^54,55^, as a composite measure comprising 17 sub-indices and numerous indicators, to assess overall country progress. Despite inherent weighting issues^56^, the SDG Index is a widely recognized and interpretable measure^57,58^. Our convergence analysis spans 2000–2023, covering 149 countries globally, offering insights into how policy efforts can be optimized for diverse country contexts and how global and national strategies can be refined to accelerate SDG attainment.

## Results

The empirical results of the club convergence analysis for global sustainable development goals are presented in Table 1. Initially, we apply the log t-test, to assess the overall convergence condition for the entire sample. For the global SDG Index, the coefficient is negative (-0.505) and the T-statistic value is -11.99, which is significantly lower than the threshold of -1.65. Therefore, the log t-test results suggest that global SDG Index does not exhibit overall convergence. The initial results of these subgroups (top panel) show 8 convergent clubs and one non-convergent club. The results of the club’s merger (test of club merging) show that, according to t-statistics, clubs 1 + 2, 2 + 3, 3 + 4, 4 + 5, and 5 + 6 can be merged, but other clubs cannot. The final club classification panel shows the results of the countries’ convergence after the merger of the clubs; as can be seen, we have 5 converging clubs and one non-converging group, given that a large number of countries are located in clubs 1 and 2. The non-converging club includes three countries: Central African Republic, Chad and Lebanon. The estimations suggest that the convergence clubs are rather weak. We can see that three of five coefficients are negative and insignificant. Only the coefficient of Club 5 is positive and significant.

**Table 1:** Convergence club results.

log(t)	Club1	Club2	Club3	Club4	Club5	Club6	Club7	Club8	Group9
Initial club classification									
Coeff	0.087	0.174	0.107	0.280	0.201	0.050	-0.004	0.412	-0.898
T-stat	1.350	2.500	1.559	3.604	2.902	0.838	-0.132	2.337	-113.024
N	30	28	27	18	12	13	14	4	3

log(t)	Club1+2	Club2+3	Club3+4	Club4+5	Club5+6	Club6+7	Club7+8	Club8+9
Test of club merging								
Coeff	-0.007	0.109	-0.032	0.109	0.043	-0.216	-0.363	-0.836
T-stat	-0.119	1.643	-0.544	1.693	0.737	-6.304	-22.973	-85.133

log(t)	Club1	Club2	Club3	Club4	Club5	Group6
Final club classification						
Coeff	-0.007	-0.032	0.043	-0.004	0.412	-0.898
T-stat	-0.119	-0.544	0.737	-0.132	2.337	-113.024
N	58	45	25	14	4	3

**Fig. 1:**
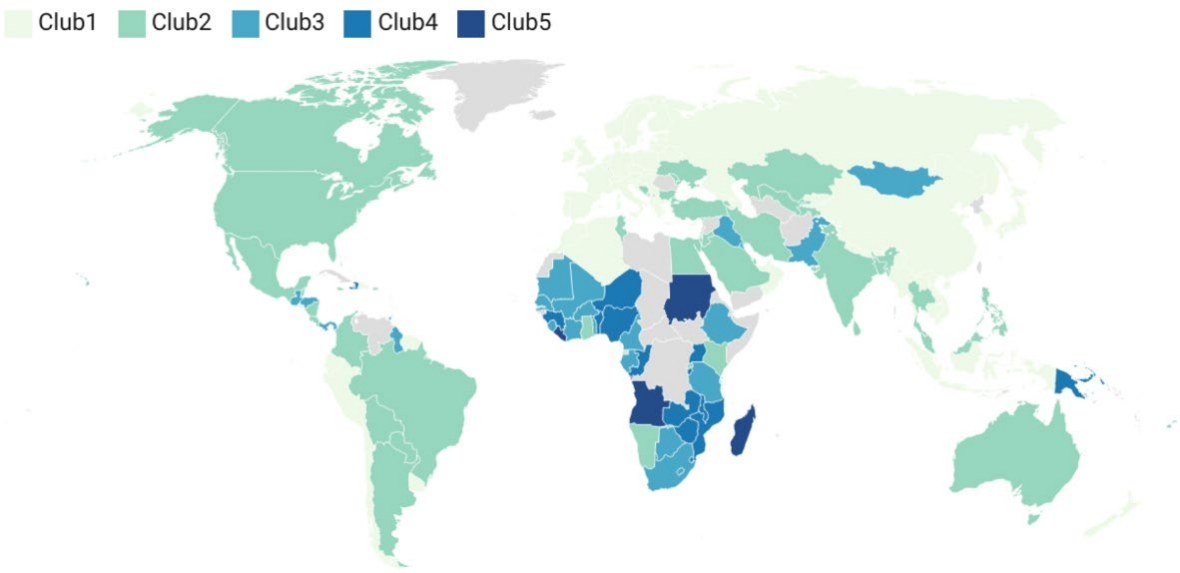
Geographical distribution of convergence clubs.

Figure 1 illustrates the geographical distribution of convergence clubs. The countries in Club 1 have achieved a high level of SDG Index, indicating strong progress and effective implementation of sustainable development practices. Countries include: Albania, Algeria, Austria, Azerbaijan, Belarus, Belgium, Bhutan, Croatia, Cyprus, Denmark, Dominican Republic, El Salvador, Estonia, Finland, France, Georgia, Germany, Greece, Hungary, Indonesia, Ireland, Italy, Japan, Korea (Rep), Kyrgyz Republic, Lao PDR, Latvia, Lithuania, Maldives, Malta, Morocco, Myanmar, Nepal, Netherlands, New Zealand, North Macedonia, Norway, Oman, Peru, Poland, Portugal, Russian Federation, Serbia, Slovak Republic, Slovenia, Spain, Suriname, Sweden, Switzerland, UAE, UK, Uruguay, Vietnam.

In Club 2, countries show a moderate level of success in achieving SDGs, indicating significant efforts and progress but with some areas needing further improvement. Countries include: Argentina, Armenia, Australia, Bangladesh, Barbados, Belize, Bolivia, Bosnia and Herzegovina, Brazil, Bulgaria, Canada, Colombia, Costa Rica, Egypt (Arab Rep), Fiji, Ghana, India, Iran (Islamic Rep), Israel, Jamaica, Jordan, Kazakhstan, Kenya, Luxembourg, Malaysia, Mauritius, Mexico, Moldova, Nicaragua, Paraguay, Philippines, Qatar, Rwanda, Saudi Arabia, Singapore, Sri Lanka, Tajikistan, Thailand, Tunisia, Turkey, Ukraine, United States, Uzbekistan.

In Club 3 countries exhibit a lower level of SDG achievement, indicating moderate progress but with substantial areas needing improvement. Countries include: Bahrain, Benin, Botswana, Brunei Darussalam, Burkina Faso, Cameroon, Gabon, Guatemala, Guyana, Haiti, Honduras, Iraq, Kuwait, Lesotho, Mali, Mauritania, Mongolia, Senegal, Sierra Leone, South Africa, Tanzania, Togo, Trinidad and Tobago.

The countries in Club 4 have an even lower SDG Index, suggesting significant challenges in their sustainable development efforts and areas requiring considerable focus and improvement. Countries included: Burundi, Congo (Rep), Eswatini, Guinea, Malawi, Mozambique, Niger, Nigeria, Papua New Guinea, Uganda, Zambia, Zimbabwe.

In Club 5 countries have achieved the lowest level of SDG Index, indicating significant challenges and a need for focused efforts to improve their efforts towards sustainable development. Countries included: Angola, Liberia, Madagascar, Sudan.

**Fig. 2:**
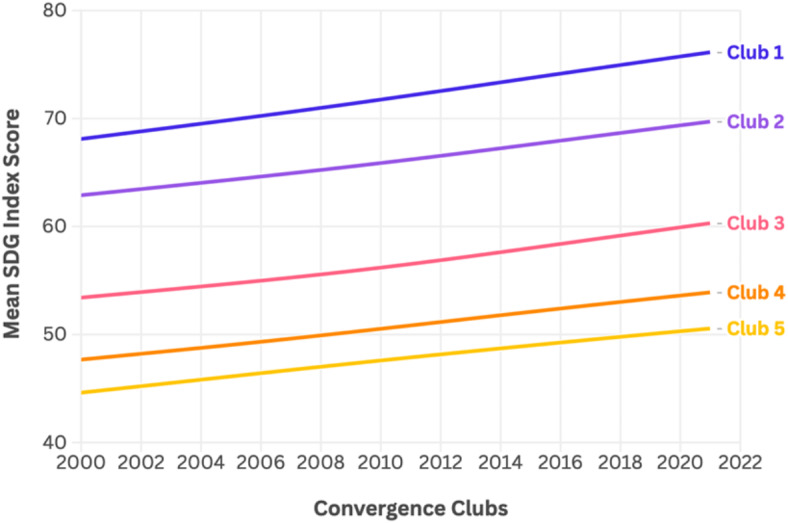
Mean of SDG Index values by convergence clubs. Note: Clubs are classified based on SDG Index performance levels: Club 1 represents high-performing countries, while Club 5 includes the lowest-performing nations.

Figure 2 shows the average SDG indexes across clubs, with the divergent countries being also represented. It can be observed that there is a similar distance among clusters in the final stage of the period, denoting clearly the absence of convergence in the entire sample. With the aim of formally exploring the determining factors of these clusters, the next step will perform an ordered logit model.

After identifying the convergence clubs, we analyze the factors that influence the SDG performance using the ordered logistic regression framework, where club membership represents the level of SDG attainment. A higher club number indicates a lower level of achievement along the SDG Index. We employ various explanatory variables utilizing different data sources. For quantifying the level of globalization of countries, we use the KOF Globalization Index, a composite index with different sub-indices in a hierarchical structure^59,60^. For income, GDP per capita is used as a proxy. For urbanization, the growth rate of urban population is used (this indicator tends to have lower values, since a large share of population already lives in urban areas and there is no more room for further growth there). The ecological footprint of countries is measured based on the data of the Global Footprint Network^61^. We estimate three different models using different aggregations of the KOF indices (Table 2). Our findings reveal that higher GDP per capita and various aspects of globalization (economic, social, political, trade, and cultural) are positively associated with SDG achievements, while urban population growth and ecological footprint are negatively associated. The analysis highlights the multifaceted nature of sustainable development and the varying impacts of globalization dimensions on the SDG Index.

**Table 2:** Ordered logistic regression results for factors influencing SDG club membership (2000–2023).

Variable	Model 1	Model 2	Model 3
ln(GDP/capita)	-0.421***	-0.285***	-0.271***
Urban population growth (UPG)	0.265***	0.237***	0.192***
Ecological footprint (EFP)	0.068***	0.077***	0.079***
KOF Globalization Index (GI)	-0.058***		
KOF Economic Globalization Index (EGI)		-0.011***	
KOF Social Globalization Index (SGI)		-0.039***	
KOF Political Globalization Index (PGI)		-0.016***	-0.016***
KOF Trade Globalization Index (TGI)			-0.036***
KOF Financial Globalization Index (FGI)			0.040***
KOF Interpersonal Globalization Index (IGI)			-0.010
KOF Informational Globalization Index (InfGI)			0.005
KOF Cultural Globalization Index (CGI)			-0.045***
N	3212	3212	3212
Pseudo R^2^	0.173	0.176	0.201
Log-likelihood	-3.6e + 03	-3.6e + 03	-3.5e + 03

Note: Significance levels are denoted as follows: ***p < 0.01, **p < 0.05, *p < 0.1.

**Fig. 3:**
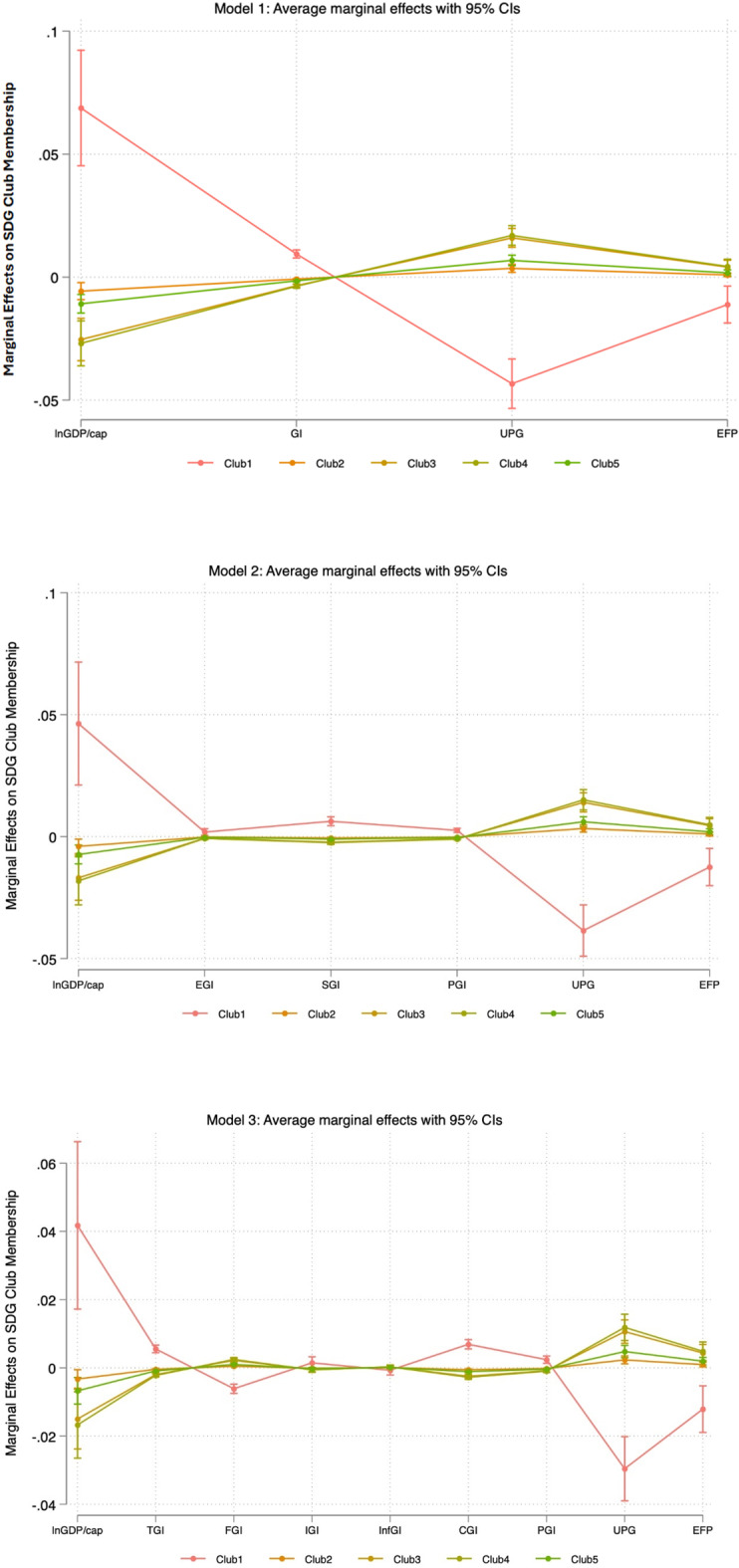
Marginal effects of the predictors from ordered logistic regression models (Models 1–3). Note: Higher bars indicate stronger impacts of predictors, such as GDP per capita, on the probability of achieving higher SDG performance (Club 1).

The marginal effects analysis from Model 1–3 reveals the significant differences between Club 1 and the other clubs in terms of sustainable development (Fig. 3). Higher GDP per capita considerably enhances the likelihood of a country achieving high SDG performance, distinguishing Club 1 from lower-performing clubs. Rapid urbanization negatively impacts the probability of being in Club 1, highlighting the challenges it poses to achieving top SDG Index outcomes. Environmental degradation in terms of ecological footprint significantly reduces the likelihood of high SDG performance. The high ecological footprint is negatively associated with membership of Club 1. Increased globalization and its components positively influence the probability of being in Club 1, showing its beneficial effects are more substantial for the highest-performing countries except financial and informational globalization.

## Discussion

This study addressed the different influences of economic, social, and environmental factors on the progress towards UN Sustainable Development Goals (SDGs), based on a club convergence framework, resulting in a classification of countries into five clubs.

The impacts of globalization on sustainable development are very complex, as highlighted by the results of our ordered logit model and marginal effects analysis. However, it seems that country groups with higher level of globalization along most aspects tend to have better achievements along sustainable development, mostly in line with several earlier findings^5,9^. However, globalization is a dual-edged sword in relationship to sustainable development. Indeed, higher levels of globalization, particularly in economic, social, and cultural dimensions, positively influence the SDG Index. This is evident in the higher likelihood of Club 1 membership for countries with higher globalization index values. Globalization facilitates the flow of knowledge, technology, and resources, contributing to sustainable development. However, not all aspects of globalization are beneficial: financial globalization, for instance, shows a complex relationship, requiring careful management to avoid improving inequalities and environmental stress.

Our findings indicate that higher GDP per capita significantly enhances the likelihood of a country achieving a higher SDG Index, particularly in Club 1. This aligns with the notion that economic prosperity provides the necessary resources for investment in sustainable development initiatives. However, the challenge remains in ensuring how economic growth can be harmonized with socially and environmentally beneficial progress^10,22^. The positive correlation between GDP per capita and the SDG Index values suggests that wealthier nations are better positioned to meet the comprehensive demands of the SDGs^23,62^, but it raises fundamental questions about the sustainability of growth-focused models in the long term^58^.

The analysis reveals that rapid urban population growth negatively impacts the probability of a country being in Club 1, emphasizing the sustainability challenge posed by urbanization. Urban areas, while being centers of economic activity and innovation, also face significant pressure related to infrastructure, transportation and resource consumption. The negative marginal effects of urban population growth on SDG Index values highlight the need for sustainable urban planning and policies that mitigate the environmental impacts of urbanization^38,40^.

The ecological footprint, as a key environmental indicator in this study, emerges as a crucial determinant of SDG performance. Countries with larger ecological footprints generally exhibit lower SDG performance, highlighting the adverse effects of resource overuse and environmental degradation. This observation partially diverges from the Environmental Kuznets Curve hypothesis, which does not hold true for developed (EU) countries^63^. However, in developing countries, the relationship between ecological footprint and SDG performance can be more diverse, potentially aligning with our findings^64^. These results emphasize the importance of reducing ecological footprints through sustainable consumption and production practices^65,66^, as well as enhancing the efficiency of resource use^67^.

The results of this study highlight the need for tailored policy interventions that address the unique challenges and opportunities faced by different country groups. For high-performing countries (Club 1), policies should focus on maintaining economic stability while promoting environmental and social sustainability. For lower-performing countries, efforts should be directed towards enhancing economic growth, managing urbanization effectively, while again, avoiding ecological deficit. The role of globalization should be harnessed to support sustainable development, ensuring that its benefits are equitably distributed and its negative impacts mitigated.

This comprehensive analysis provides complex insights into the determinants of sustainable development with a special focus on the role of globalization. While economic growth and globalization seem to offer pathways to have more successful efforts to achieving SDGs, the challenges of urbanization and ecological sustainability must be addressed to ensure holistic and long-term progress. Policymakers must adopt a multifaceted approach, balancing economic, social, and environmental priorities to accelerate progress towards the UN SDGs and create a sustainable future for all.

### Challenges and future research directions

There are several challenges and potential future improvement options to further improve the robustness and applicability of the approach suggested in this study. The current framework uses the SDG Index, a composite measure with weighting issues, even if the index itself is widely recognized. Indeed, a limitation of this study is that sustainability, a very complex and multi-dimensional concept is measured with one – even though composite – index, the SDG Index, leading to unavoidable simplifications^56,62^. Future research on modelling sub-indices of the main index, or even specific SDG indicators may provide more detailed results for the respective fields.

Another direction of improvement may be the integration of additional – hard to be quantified – contextual factors, such as governance quality, level of environmental regulations and geopolitical stability, which may significantly influence performance along SDGs. Expanding the dataset to include temporal lag effects or interactions between variables, such as the interplay between urbanization and globalization dimensions, could provide deeper insights. Moreover, refining the club convergence methodology itself by using spatial econometric techniques could give the chance for the examination of regional spillover effects and interdependencies among countries, providing a more comprehensive perspective on global and regional dynamics in relationship to sustainability.

Furthermore, by applying alternative methods, such as dynamic panel models, deep or machine learning techniques that also emerge in the field of sustainable development^68,69^, the non-linear relationships and interactions between explanatory variables could be better explored.

Related to challenges of the dataset, the temporal coverage (2000–2023) hinders to fully capture long-term trends, especially for countries with incomplete data for earlier years, so future studies could address this limitation by employing robust imputation techniques or focusing on datasets with more comprehensive historical records.

Another challenge is related to the heterogeneity of data sources used in this study. Indicators such as the ecological footprint and the KOF Globalization Index are derived from diverse methodologies, with a threat of inconsistencies. Data availability is also an issue: the components of globalization dimensions were analyzed extensively, other relevant factors, such as cultural or institutional differences, were not included, so the expansion of the dataset to incorporate additional socio-political and environmental indicators could enhance the robustness and generalizability of the findings, thus addressing these challenges will be critical for improving the reliability of future research and advancing understanding in this field.

### Data and methods

In the first step of the club convergence model approach, we utilize the log t-test^50^ to assess the convergence patterns globally and evaluate the convergence in log SDG Index across different nations. The findings in the literature^50^ demonstrate that the assumption of a homogeneous technological process introduces inconsistencies in classical β-convergence due to omitted variable bias and endogeneity issues. To address this, the authors proposed an alternative methodology, the log t-test, in order to capture various convergence types observed in empirical settings. This test, in relationship to the modification of the neoclassical growth model, grabs transitional cross-sectional divergence by allowing model parameters to vary across sections. Importantly, the model assesses overall relative convergence among cross-sections, with alternatives permitting both overall divergence and club convergence within regional subgroups.

Formally we can describe this approach the following way. The first step involves calculating the relative transition parameter, which measures the relative position of each unit (e.g., country) with respect to the group’s average at each point in time. This parameter is defined as:

1 $$\:{h}_{it}=\frac{{y}_{it}}{\frac{1}{N}\sum\:_{i=1}^{N}{y}_{it}}$$

where y_it_ represents the variable of interest (e.g. food price inflation) for country i at time t, and N is the number of countries in the sample, and the transition parameter h_it_ allows assessing the relative dynamics, focusing on the tendency of individual units to converge toward a common pattern.

The log t-test is then applied to assess if different units converge to a common steady-state path. The test involves the estimation of the following regression:

2 $$\:\text{log}\left(\frac{{H}_{1}}{{H}_{t}}\right)-2loglog\left(t\right)=\alpha\:+\beta\:\text{log}\left(t\right)+{\epsilon\:}_{t}$$

where $\:{H}_{t}=\frac{1}{N}\sum\:_{i=1}^{N}{\left({h}_{it}-1\right)}^{2}$ The key parameter of interest is $\:\widehat{\beta\:}$: if $\:\widehat{\beta\:}\ge\:0$, we fail to reject the null hypothesis of convergence, implying that the series converge. A significantly negative $\:\widehat{\beta\:}$ indicates divergence.

If overall convergence is rejected, Phillips and Sul’s method proceeds with a clustering algorithm to identify convergence clubs. The algorithm iteratively forms groups (clubs) by maximizing the homogeneity within each club, applying the log t test within subgroups. This step helps to identify distinct clusters that converge to different steady-states, addressing heterogeneity in convergence behavior across units.

Next, we investigate the characteristics that differentiate the emerging convergence clubs with the employment of ordered logistic regression analysis. This model is proper when the dependent variable is ordinal—in this case, club membership levels that have a natural order (e.g., Club 1, Club 2, …, Club k). Unlike linear regression, the ordered logistic regression considers the ordinal nature and ensures that predicted probabilities fall within the [0, 1] interval. It allows us to model the relationship between the likelihood, if a certain unit (country) belongs to a higher or lower convergence club and various explanatory variables.

Marginal effects were then calculated to strengthen the interpretability of the results of the regression. These quantify the change in the probability if a country is classified into a specific club depending from a one-unit change in each predictor, when other variables are kept unchanged.

The coefficients of the model show evidence on the direction and strength of the relationship between the predictors and the likelihood of belonging to a different (higher or lower) convergence club. A positive coefficient value shows that an increase in the predictor is in relationship to higher odds of being in a higher-numbered club (lower convergence). Similarly, a negative coefficient indicates higher odds of being in a lower-numbered club (higher convergence).

We developed three logistic regression models in order to explore variations in the influence of globalization dimensions:

#### Model 1

Includes the overall KOF Globalization Index as a single explanatory variable.

#### Model 2

Uses disaggregated globalization sub-indices (economic, social and political sub- dimensions).

#### Model 3

Sub-indices of globalization are split into even smaller sub-areas, such as the globalization of trade, financial, cultural and informational globalization.

Each model has the idea to test the incremental explanatory power of disaggregated globalization components in influencing the SDG Index. When these models are compared, the study identifies specific dimensions of globalization that have significant relationship with sustainable development progress (in terms of the SDG Index).

### Data

The study analyzes data from 149 countries over 23 years (2000–2023), resulting in a total of 3,212 observations. The dataset includes composite SDG Index scores, GDP per capita, urban population growth rates, ecological footprints, and KOF Globalization Index sub-components. The SDG Index data are retrieved from the SDG Index database^70^, with a temporal coverage from 2000, providing the timeframe of this study, too. The GDP data, as well as the urban population growth data were obtained from World Bank’s World Development Indicators database^71^. Ecological footprint data were gained from the Global Footprint Database^61^. For measuring globalization, the KOF Globalization Index database^60^ was used (including data for sub-indices – economic, social and political globalization – and further sub-categories: trade, financial, interpersonal, informational and cultural globalization.

## Data Availability

Data are obtained from public databases, as cited in the manuscript. Upon request, the authors are glad to share the database, built on the raw data. Please contact one of the authors, Imre Ferto: imre.ferto@uni-corvinus.hu.
